# A Phase I, Open-Label, Dose-Escalation Study to Evaluate the Safety and Tolerability of Intravenous UMC119-06 in Patients with Acute Ischemic Stroke

**DOI:** 10.7150/ijms.124255

**Published:** 2026-01-01

**Authors:** Lung Chan, Jia-Hung Chen, Chien-Tai Hong, Yu-Cheng Lin, Jui-Hung Chang, Chien-Chang Chen, Yogi Cheng-Yo Hsuan, Chaur-Jong Hu

**Affiliations:** 1 Department of Neurology, school of medicine, college of medicine, Taipei Medical University, Taipei, Taiwan.; 2 Department of Neurology, Taipei Medical University - Shuang Ho Hospital, New Taipei, Taiwan.; 3 Meribank Biotech Co., Ltd., Taipei 114, Taiwan.; 4 Reon Biotech Co., Ltd., Taipei 114, Taiwan.

**Keywords:** ischemic stroke, mesenchymal stem cell, biomarkers, dose-escalation study

## Abstract

This first-in-human, open-label, dose-escalation study (NCT04097652) evaluates UMC119-06, a mesenchymal stem cell product derived from human umbilical cord, for the treatment of acute ischemic stroke (AIS). The study includes two dosing cohorts: 1×10^6 cells/kg and 5×10^6 cells/kg, with three participants enrolled in each cohort. The primary objective is to assess the safety, tolerability, and maximum feasible dose of UMC119-06 in AIS patients, while the secondary objective focuses on evaluating long-term safety and clinical efficacy following a single administration. The study conducted safety assessments across both dosing cohorts, demonstrating that UMC119-06 is well-tolerated, with no adverse events (AEs) classified as possibly, probably, or definitely related to the product. Despite the small sample size of six patients, trends toward positive outcomes were observed in the modified Rankin Scale, the National Institutes of Health Stroke Scale, the Barthel Index, and the infarct volume on brain magnetic resonance imaging. Additionally, changes in biomarkers such as IL-6, TNF-α, VEGF, and HGF suggest a potential impact of UMC119-06 on inflammation, angiogenesis, and tissue repair processes. This Phase I study provides preliminary evidence regarding the safety and potential efficacy of UMC119-06 in AIS patients. Larger-scale studies with control groups are warranted to validate these findings and further explore the therapeutic potential of UMC119-06 for AIS.

## Introduction

Stroke remains a leading cause of death and long-term disability worldwide, with its burden rising over the past decade 1. Despite advancements in early intervention therapies, such as tissue plasminogen activator and mechanical thrombectomy, treatment options remain limited, and many patients continue to experience long-term neurological deficits. Stem cell-based therapies, with the potential to promote tissue regeneration, repair damaged neurons, and restore lost functions through the stimulation of neurogenesis and angiogenesis, are emerging as a promising approach for stroke treatment 2,3. Recent studies have shown that stem cells can migrate to damaged areas of the brain, differentiate into neural cells, and integrate into existing neural circuits, potentially improving functional recovery 4,5.

Mesenchymal stem cells (MSCs), one of the most commonly used types of stem cells, have demonstrated potential for stroke recovery in preclinical studies 6,7,8. Among MSCs, umbilical cord-derived MSCs (UCMSCs) have gained increased attention due to their ease of isolation, low immunogenicity, and powerful regenerative capabilities 9,10. UCMSCs can migrate to injury sites through chemokine-mediated homing and promote tissue repair through paracrine signaling 11. These cells exert therapeutic effects by modulating inflammatory responses, enhancing angiogenesis, and promoting neurogenesis, which collectively contribute to functional recovery after stroke 12,13,14. Furthermore, UCMSCs have a favorable safety profile, making them a promising candidate for clinical application 15,16.

Despite promising preclinical findings, few clinical trials have explored the therapeutic application of UCMSCs in acute ischemic stroke (AIS). The heterogeneity of stroke pathology, variation in treatment protocols, and challenges in standardizing cell-based therapies have hindered progress in this field. To address this unmet need, we initiated a phase I, first-in-human, open-label, dose-escalation study to evaluate the safety and tolerability of UMC119-06, a UCMSCs product, in AIS patients. The primary objectives were to assess the safety, tolerability, and maximum feasible dose (MFD) of UMC119-06. The secondary objectives included evaluating long-term safety and preliminary clinical efficacy following a single intravenous administration of UMC119-06. Additionally, the study investigated the effects of UMC119-06 on key stroke-related biomarkers, which could offer insights into its mechanisms of action and potential therapeutic benefits. Ultimately, the study aimed to provide foundational data to guide future investigations of UCMSC-based therapies for AIS.

## Method

### Protocol approvals, registration, and informed patient consent

The UMC119-06 trial protocol (ClinicalTrials.gov identifier: NCT04097652) was approved by the Taiwan Food and Drug Administration (No. 1086027359) and the Institutional Review Board of Taipei Medical University - Shuang Ho Hospital (N201904089). The study was conducted in accordance with the Declaration of Helsinki, and informed consent was obtained from all participants or their legally authorized representatives following a thorough explanation.

### Trial design and oversight

**Figure 1:**
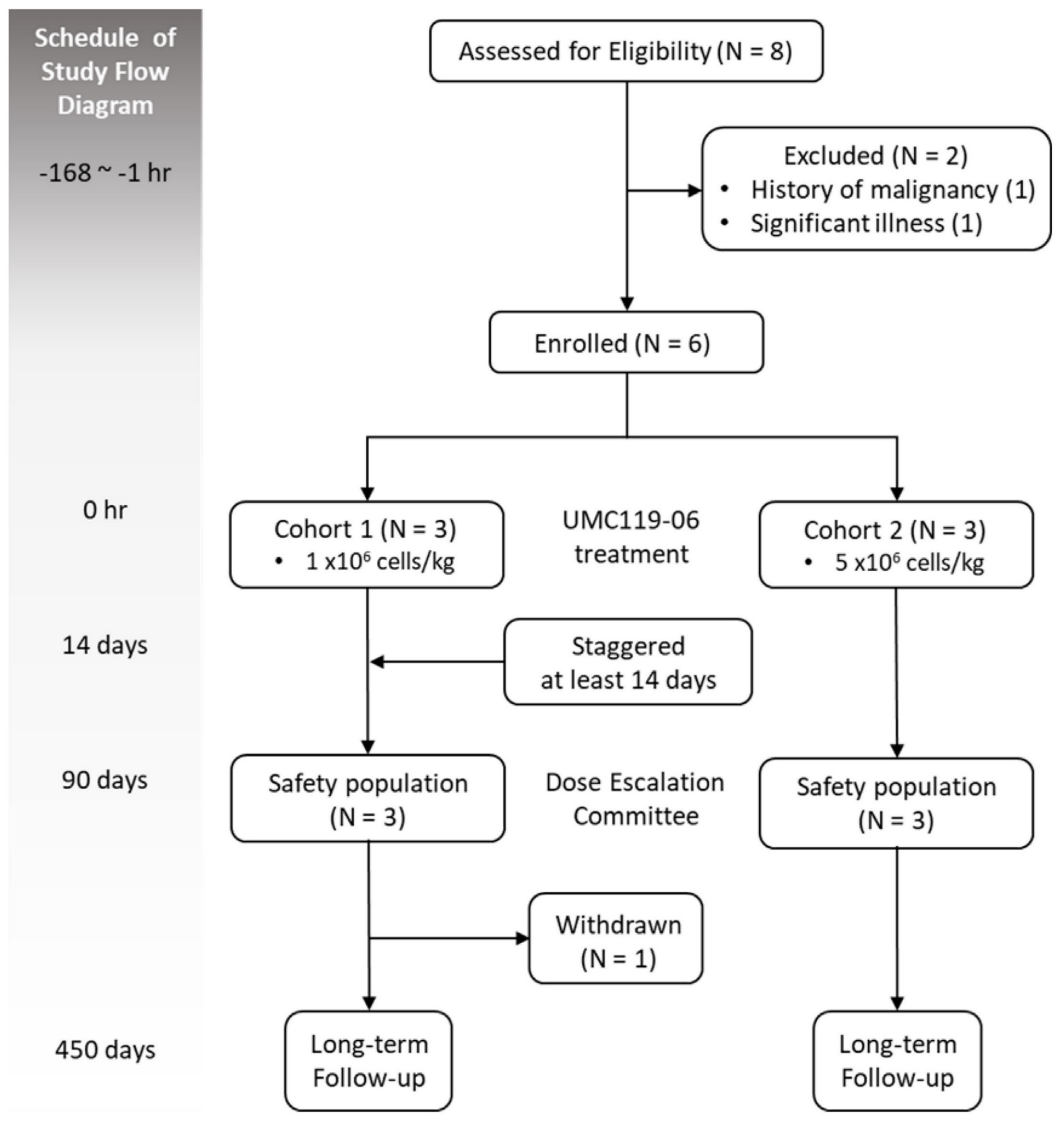
** Study Diagram for Trial Design and Oversight**.

This phase I, open-label, dose-escalation trial was designed to evaluate the safety, tolerability, and MFD of UMC119-06 in patients with AIS. The study employed a standard 3 + 3 dose-escalation design, with sentinel dosing at each dose level to determine the MFD. Escalation to the next cohort was permitted if 0 out of 3 patients or ≤ 1 out of 6 patients experienced dose-limiting toxicity (DLT). Enrollment would be discontinued if ≥ 2 patients experienced DLT. The trial design flowchart is shown in Figure 1.

UMC119-06 is a MSC derived from human umbilical cords of pregnant women, manufactured in a Good Tissue Practice (GTP) laboratory for cell therapies (Reon Biotech Co., Taiwan). It was formulated in 0.9% normal saline with 2% clinical-grade human serum albumin, with a cell density ranging from 3.3×10⁵ to 2.3×10⁶ cells/mL. Dose selection for the human study was determined based on preclinical animal experiments in which three dosage levels were evaluated. The low-dose group received a mean dose of 3.42×10⁵ cells per rat, the mid-dose group received 0.95×10⁵ cells per rat, and the high-dose group received 3.18×10⁶ cells per rat, with no significant safety issues observed. To establish an appropriate clinical dosing strategy, standard allometric scaling considerations were applied, accounting for interspecies differences in body weight, expected cell distribution, and the viability and potency of the final cell product. In this study, eligible patients were assigned to either cohort 1 (1×10⁶ cells/kg) or cohort 2 (5×10⁶ cells/kg) for a single intravenous infusion of UMC119-06.

### Participants

Eligible patients were aged between 20 and 80 years, with a stroke onset within 48 to 168 hours before the start of treatment, and the stroke was caused by large-artery atherosclerosis or cardioembolism. Patients were required to have a pre-treatment modified Rankin Scale (mRS) score of 0 or 1, National Institutes of Health Stroke Scale (NIHSS) scores between 5 and 20, and no increase of ≥4 points in NIHSS scores from baseline. The presence of a hemispheric cortical infarct was confirmed by brain magnetic resonance imaging (MRI), with a lesion size of less than 100 mL on diffusion-weighted imaging. Exclusion criteria included patients with hemorrhagic transformation on computed tomography (CT) scan, lacunar or brainstem infarct, seizure attack, or significant head trauma (defined as a Glasgow Coma Scale score of 3 to 8). Additionally, those with uncontrolled hypertension, uncorrected coagulopathy, a history of malignancy, major surgery within the past 30 days, pregnancy, HIV infection, or significant illness were also excluded. Complete details of the inclusion and exclusion criteria for patient eligibility during screening are provided in the supplementary material (Table S1).

### Trial process

The trial was conducted at Taipei Medical University - Shuang Ho Hospital in Taiwan. Eligible patients were assigned to either cohort 1 or cohort 2. Baseline characteristics and vital signs were recorded before the administration of UMC119-06. Patients received a single-dose IV infusion of UMC119-06 during the treatment phase. A physical examination was performed 30 minutes after the infusion was completed. To mitigate any potential safety risks, patients were hospitalized for 14 days during the DLT evaluation period. Safety data from the first patient in each cohort, as well as from all patients in completed cohorts, were reviewed by the Dose Escalation Committee (DEC) before further patient enrollment or dose escalation. No interim efficacy analysis was planned for this trial. Clinical assessments were conducted within 3 months post-treatment, with an additional 1-year safety follow-up (Figure 1).

### Outcomes assessment

The primary outcomes of this trial were the safety, tolerability, and MFD of UMC119-06 in patients with AIS. The safety and tolerability of UMC119-06 were assessed throughout the study by monitoring vital signs, clinical laboratory data, electrocardiograms, physical examinations, and adverse events (AEs)/treatment-emergent adverse events (TEAEs). The MFD of UMC119-06 was determined based on the occurrence of DLTs among TEAEs. The involvement of the DEC in reviewing data from each cohort ensured the trial's rigor and safety oversight.

The secondary outcomes of this trial were improvements in clinical function, as assessed by changes in mRS scores, NIHSS scores, Barthel Index (BI) scores, and infarct volume on brain MRI. The mRS and BI were used to measure the degree of disability or dependence in daily activities, while the NIHSS was used to quantify the stroke-related impairment. Infarct volume was measured on brain MRI using RAPID software to assess recovery in all patients, with apparent diffusion coefficient (ADC) values < 620 defined as the infarcted area.

### Adverse events

According to 21 CFR 312.32(a), an AE is defined as any unfavorable or unintended sign, symptom, or disease that occurs, is reported by the patient, or represents a worsening of a pre-existing condition. An AE may or may not be related to the study treatment. Any abnormal physical examination finding or laboratory result that the investigator considers clinically significant to the patient and that occurs after the initiation of the first trial treatment will be reported as an AE.

### Statistical analysis

There was no planned inferential statistical analysis in this trial. However, exploratory inferential statistical tests may be conducted, with a two-sided significance level of 0.05. All safety assessments will be descriptive. Statistical analyses were performed using the Statistical Analysis System (SAS)® for Windows, version 9.4 or later. Outcome analysis images will be generated using GraphPad Prism 6, with data summaries presented according to the variable type (continuous and categorical).

## Results

### Patient characteristics

A total of 8 patients were screened during the trial, of whom 2 did not meet the eligibility criteria, resulting in the enrollment of 6 patients. These patients were sequentially assigned to one of the two dose cohorts, with 3 patients in each cohort. One patient in cohort 1 withdrew early upon the principal investigator's suggestion. A total of 5 patients completed the study (Figure 1). All 6 enrolled patients were included in the safety population. Of the 6 patients, 4 (66.7%) were female. The mean age was 64.0 ± 9.8 years, and the mean body weight was 64.4 ± 8.7 kilograms. All patients had a history of hypertension; 4 had diabetes mellitus, 5 had dyslipidemia, 1 had atrial fibrillation, and 2 had a prior stroke. The median NIHSS score was 9.5 (range 5-17) (Table 1).

### Safety outcomes

The safety of UMC119-06 was evaluated across two dose cohorts: an initial dose of 1 × 10⁶ cells/kg and a high dose of 5 × 10⁶ cells/kg. Of the 6 patients, 5 (83.3%) experienced AEs or TEAEs, with 4 (66.7%) reporting serious adverse events (SAEs) (Table 2). No DLTs were observed during the trial. In total, 40 AEs, 35 TEAEs, and 10 SAEs were reported (Supplement S2). All TEAEs were deemed unlikely or unrelated to UMC119-06 treatment, and none of the SAEs were considered definitely, probably, or possibly related to the investigational product. Most SAEs were related to infections and respiratory complications, including COVID-19, pneumonia, empyema, septic shock, acute respiratory distress syndrome, and respiratory failure (Table 3). No deaths were reported during the trial. All abnormalities in laboratory measurements or physical examinations were deemed to be reasonably explained by an AE and were considered unlikely or unrelated to UMC119-06. Overall, no significant findings were observed in the safety evaluation described above.

### Efficacy outcomes

**Figure 2:**
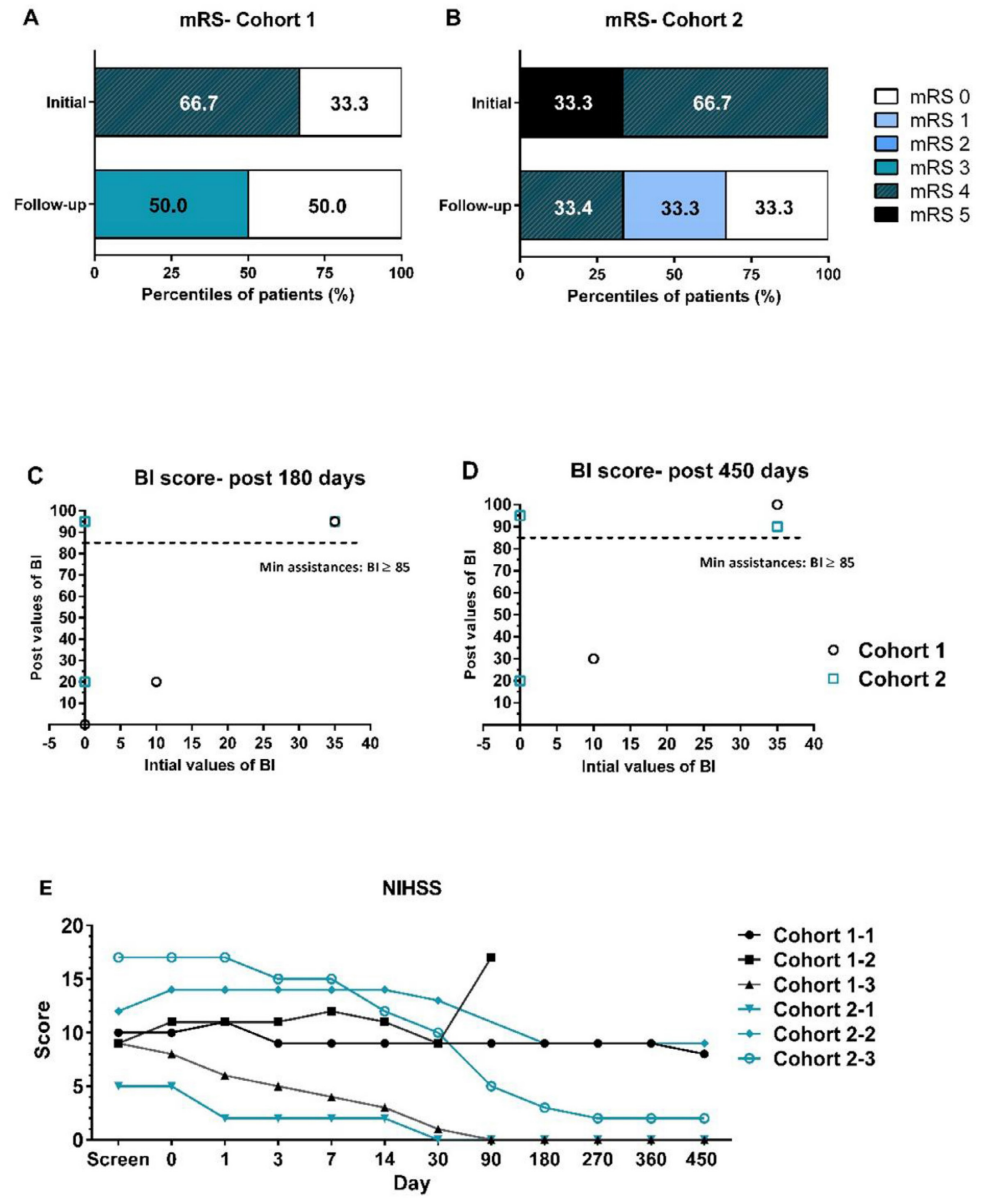
** Clinical Function Assessments by mRS, NIHSS, and BI Scores.** (A, B) Distribution of mRS scores in cohort 1 and cohort 2 at baseline and at the end of the follow-up visits. (C, D) BI score values of all patients at initial screening compared to post-treatment at 180 and 450 days. (E) NIHSS assessments of all patients throughout the trial. mRS = modified Rankin Scale; BI = Barthel Index; NIHSS = National Institutes of Health Stroke Scale.

The efficacy of UMC119-06 was assessed by evaluating clinical function using mRS, NIHSS, BI, and brain MRI. During enrollment, most patients had mRS scores ranging from 4 to 5. However, significant improvements were observed in subsequent visits. By day 450, at the end of the follow-up, 2 subjects had mRS scores of 4 and 3, while all other subjects had scores of 0 or 1 (Figure 2A and 2B). When compared to day 0, the BI scores of patients in both cohort 1 and cohort 2 reached between 85.0 and 100.0 by days 180 and 450, indicating near-complete independence for these patients (Figure 2C and 2D). Notably, the mean values for cohort 2 were higher than those for cohort 1 at all visits. Furthermore, the NIHSS scores of all patients ranged from 5 to 17 at screening, with most showing a decrease of more than 3 points from baseline starting at day 30 (Figure 2E). A trend of greater NIHSS score reduction was observed in cohort 2 compared to cohort 1.

**Figure 3:**
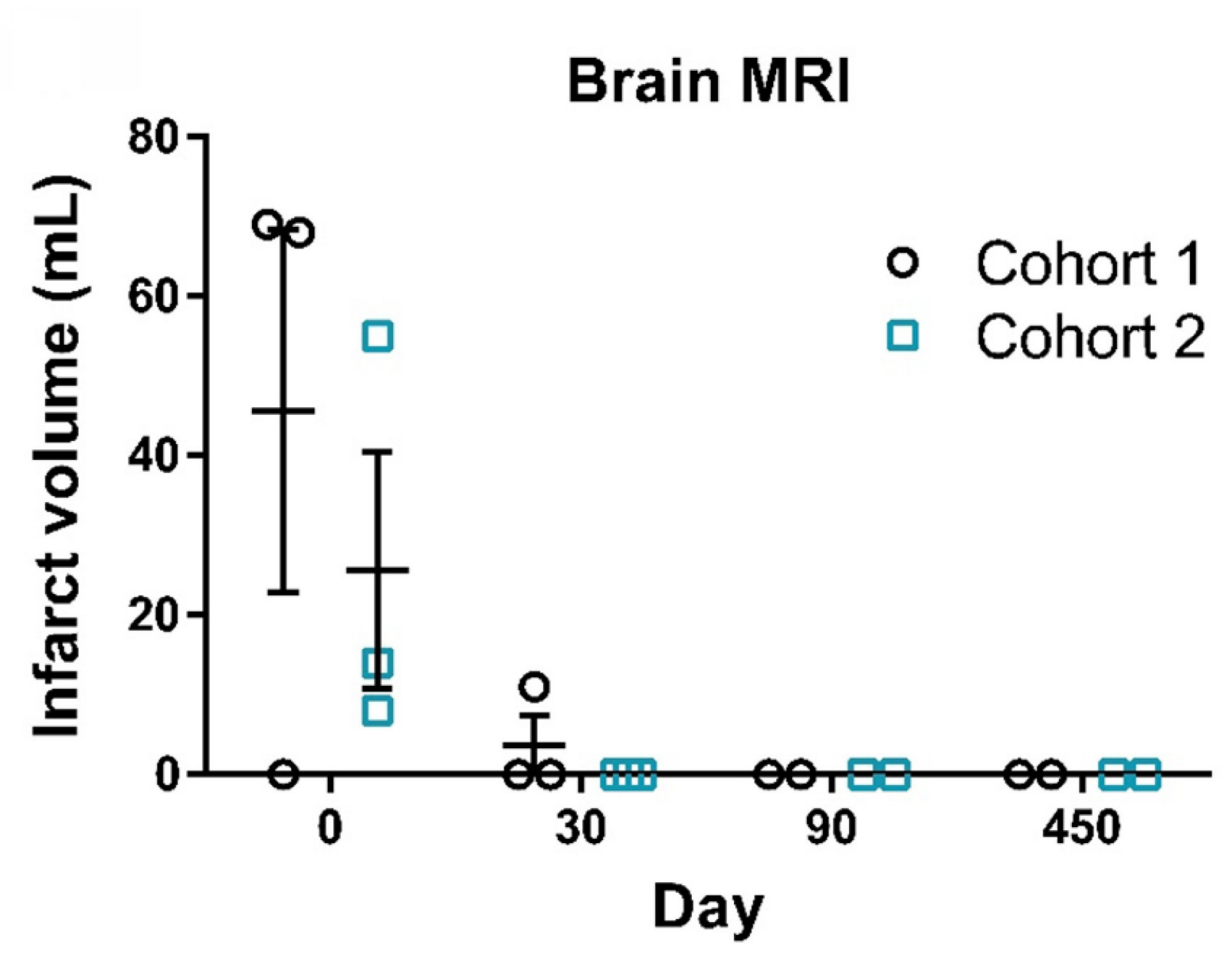
** Infarct Volume Measurement on Brain MRI.** Representative MRI images of brain infarct volumes in patients from screening to day 450. Infarct volumes of patients at screening (day 0), post-treatment (day 30 and 90), and follow-up (day 450) visits. MRI = Magnetic Resonance Imaging.

Additionally, brain MRI was performed to measure the infarct size in AIS patients following UMC119-06 treatment. The imaging showed a significant reduction in infarct volume after treatment with UMC119-06. Notably, 5 subjects had an infarct volume of 0 mL by day 30, while the remaining subject achieved 0 mL by day 90 and maintained this result thereafter, indicating favorable recovery following treatment (Figure 3). Further details on the outcome analyses are provided in Supplements S3-S6.

### Exploratory outcomes

**Figure 4:**
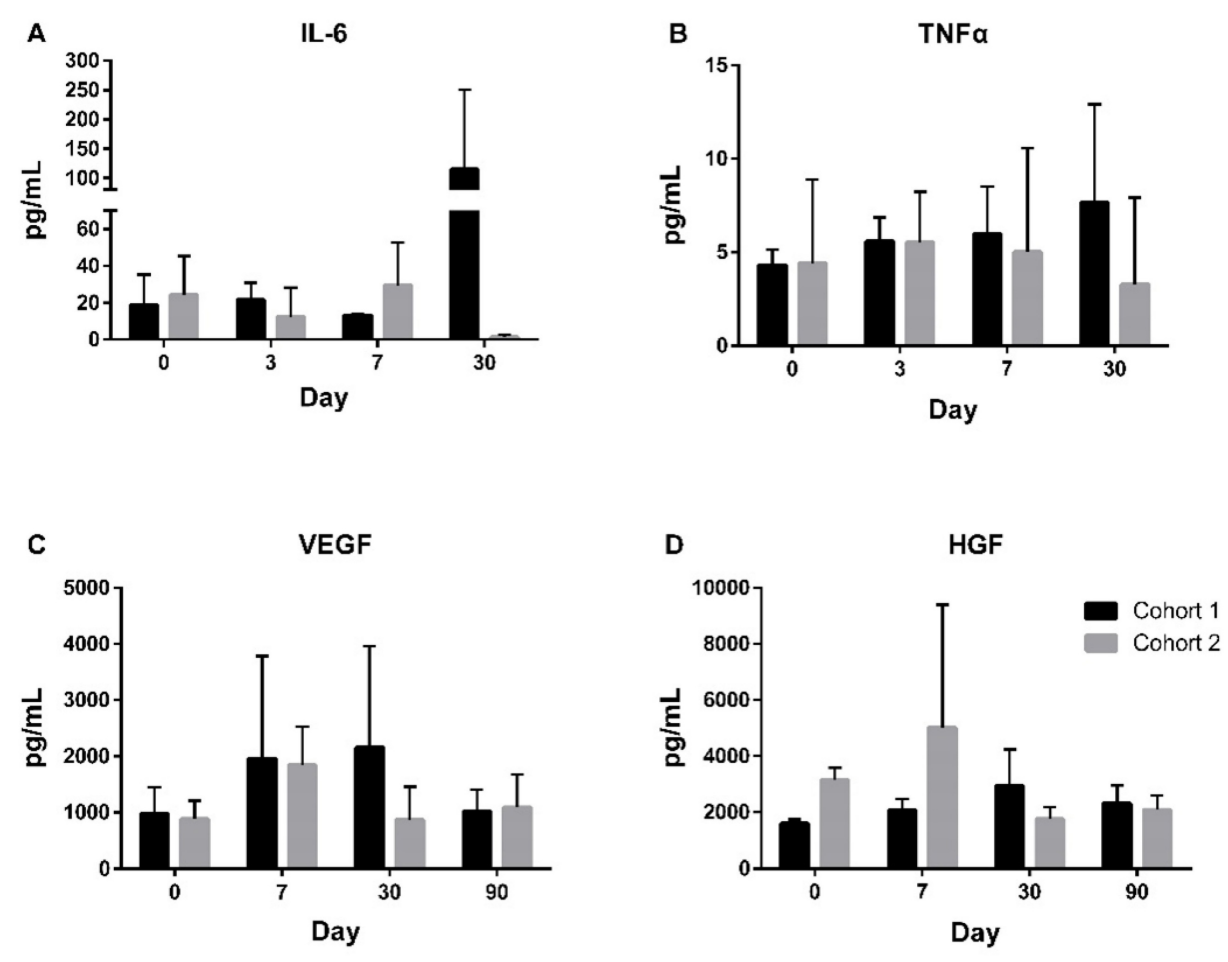
** Exploratory Biomarkers Throughout the Trial.** Exploration of biomarkers in patients from day 0 to 30 for IL-6 (A) and TNFα (B), and from day 0 to 90 for VEGF (C) and HGF (D). IL-6 = Interleukin-6; TNFα = Tumor Necrosis Factor α; VEGF = Vascular Endothelial Growth Factor; HGF = Hepatocyte Growth Factor.

AIS-related biomarkers, including interleukin-1 beta (IL-1β), interleukin-6 (IL-6), interferon gamma (IFN-γ), tumor necrosis factor alpha (TNF-α), vascular endothelial growth factor (VEGF), and hepatocyte growth factor (HGF), were evaluated throughout the trial. Among these, IL-1β and IFN-γ were mostly undetectable. From day 0 to day 30, an increase in IL-6 was observed, with a mean increase of 24.8 pg/mL (Figure 4A), while TNF-α showed only a slight increase of 0.8 pg/mL during the same period (Figure 4B). Both VEGF and HGF levels peaked on day 7 and subsequently decreased over time (Figure 4C and 4D). The mean change in VEGF from day 0 to day 90 increased by 50.2 pg/mL, while HGF levels decreased by 40.9 pg/mL.

## Discussion

The results of this Phase I trial demonstrated that UMC119-06 was well tolerated in AIS patients, with no DLTs observed. While the safety evaluation revealed a high incidence of SAEs in both dose cohorts, none of the SAEs were directly attributed to UMC119-06. The occurrence of TEAEs was also notable, though most were of mild to moderate severity. Importantly, no significant immunogenic reactions, thromboembolic events, or organ dysfunction directly linked to UMC119-06 administration were observed. These findings are consistent with previous studies that support the general safety of UCMSC therapy across various diseases 17,18,19.

The incidence of AEs was comparable between the two dose cohorts, suggesting no clear dose-related trend in event frequency. SAEs, including COVID-19, pneumonia, empyema, septic shock, and acute respiratory distress syndrome, were observed; however, most of these events are common in post-stroke patients and were considered unlikely to be related to the treatment. Nervous system-related SAEs, such as seizure and vocal cord paralysis, were observed in one patient from each cohort, but these events may also be attributable to underlying cerebrovascular disease. Overall, although AEs and SAEs were frequent, their nature and distribution align with expected complications in patients with significant baseline neurological and medical comorbidities. These findings support the continued clinical development of the investigational treatment.

In preliminary efficacy analyses, a trend toward improvement in clinical outcomes, as assessed by mRS, NIHSS, and BI, was observed in this study. Most patients had mRS scores return to 0 or 1 by day 450 post-treatment, with BI scores reaching 85 to 100, indicating that functional independence was achieved in these patients. The reduction in infarct volume on brain MRI further supports the potential neuroprotective and reparative effects of UCMSCs. These promising trends observed in clinical improvements suggest that UCMSCs may play a role in enhancing recovery and supporting functional independence in AIS patients.

The findings of this study align with a growing body of evidence suggesting that MSC therapy is safe and holds promise for improving outcomes in patients with AIS. Previous randomized controlled trials have demonstrated that autologous MSC treatment is both feasible and effective in improving motor recovery in stroke patients 20,21. However, this effect has not been observed in patients with chronic stroke, highlighting the importance of timing in MSC therapy and suggesting that early intervention may be crucial for maximizing the therapeutic benefits of MSCs in stroke recovery 22. Notably, a phase II study using allogeneic adipose tissue-derived MSCs did not meet its efficacy endpoints, although a trend toward improvement in NIHSS scores was observed in the MSC treatment arm 23. These inconsistent findings across studies suggest that while MSCs may be beneficial for stroke recovery, the origin of the MSCs may influence the efficacy of the treatment.

Unlike autologous MSCs, this study utilized UCMSCs, which are derived from the younger tissue of the umbilical cord and are thought to have greater potential for self-renewal and differentiation compared to MSCs from older tissues, such as bone marrow 24,25. UCMSCs are also less likely to be recognized as foreign by the recipient's immune system, potentially reducing the risk of immune rejection when used in therapies, especially in allogeneic transplantation 26. Recent studies using allogeneic MSCs for stroke treatment have not shown improvements in short-term outcomes, further suggesting that UCMSCs may offer a more feasible option for stem cell-based therapies 27. However, additional studies with larger sample sizes and longer follow-up periods are needed to fully assess the efficacy and safety of UCMSCs in stroke treatment.

The therapeutic potential of MSCs in stroke recovery is mediated through several mechanisms. MSCs secrete a variety of growth factors and cytokines, including brain-derived neurotrophic factor (BDNF), VEGF, and HGF, all of which promote neuronal survival and repair 28,29. These factors help mitigate ischemic damage by promoting cell survival, reducing inflammation, and enhancing tissue repair in the affected brain regions. Additionally, MSCs can differentiate into neuronal and glial cells, offering a potential mechanism for replacing damaged tissue 30. The immunomodulatory properties of MSCs also play a crucial role in suppressing excessive inflammation by releasing anti-inflammatory cytokines, which helps prevent secondary damage 31. Furthermore, the angiogenesis process induced by MSCs promotes tissue healing and functional recovery through restoring cerebral blood flow, a key factor in the repair and regeneration of brain tissue 32,33.

To explore the potential mechanism underlying UMC119-06 in stroke treatment, this study examined changes in stroke-related biomarkers to better understand its biological effects. The elevated levels of IL-6 observed on day 30 post-treatment raise important considerations regarding the immune response and the long-term effects of stem cell therapy after ischemic stroke. The sustained IL-6 levels might indicate that MSCs are stimulating certain immune pathways that are necessary for tissue repair but also contributing to chronic low-level inflammation 34. TNF-α, another key inflammatory cytokine, exhibited only slight changes, suggesting that its role in the inflammatory response may be less pronounced in the context of UMC119-06 treatment. The combined effects of IL-6 and TNF-α levels provide important insight into how UMC119-06 influences immune modulation in stroke recovery.

Angiogenic and neurotrophic factors, including VEGF and HGF, showed significant changes following treatment. VEGF levels peaked on day 7 post-treatment and then gradually decreased, reflecting its role in promoting angiogenesis and supporting vascular remodeling in the ischemic brain. The transient surge in VEGF suggests that UMC119-06 may contribute to neovascularization during the early post-stroke recovery phase. Similarly, HGF, a key factor in neuroprotection and tissue repair, increased shortly after treatment and then declined, indicating its involvement in the initial stages of post-stroke recovery. These biomarker changes also provide valuable mechanistic insights into the potential therapeutic benefits of UMC119-06 therapy for patients with AIS, particularly through the angiogenesis process, which plays a crucial role in tissue repair and functional recovery after stroke.

Overall, UMC119-06 exhibited a favorable safety profile and potential therapeutic efficacy in the treatment of AIS. Its effects are likely mediated through a combination of immunomodulation, enhanced angiogenesis, neuroprotection, and secretion of regenerative paracrine factors, supporting its promise as a multifaceted therapy. However, there are several limitations that should be addressed. First, the study enrolled only six patients, which limits its statistical power and the generalizability of the findings. A larger sample size would provide more reliable data, allowing for stronger conclusions regarding the safety and efficacy of UMC119-06 in AIS patients. Second, the open-label design of this study introduces the potential for bias in outcome assessments. Without a control group, it is difficult to definitively attribute the observed clinical improvements to the treatment, as investigator expectations may influence the results. Third, the study did not control for other variables that might impact recovery, such as patients' medical histories, concurrent medications, or the timing of treatment relative to stroke onset. These factors could all contribute to clinical improvements, making it difficult to isolate the specific effects of UMC119-06.

In summary, challenges persist in translating MSC-based therapies into clinical practice. Key considerations include the standardization of cell manufacturing, determination of optimal cell dosages, and long-term safety assessments. Exploring the precise mechanisms through which MSCs mediate neuroprotection and functional recovery will be crucial for refining treatment strategies. The biomarker findings from this study highlight the importance of monitoring inflammatory and regenerative markers to better understand the biological effects of UCMSC therapy, which could inform and guide future clinical applications.

## Conclusion

This Phase I study provides initial evidence supporting the safety and tolerability of UMC119-06, a UCMSC-derived therapy, in patients with AIS. While preliminary efficacy signals were observed, larger-scale, randomized trials are needed to confirm the therapeutic benefits and determine the optimal clinical application of UCMSC therapy for stroke. Given the unmet clinical need for effective stroke treatments, UCMSC-based interventions show promise as a potential approach to enhance neuroprotection and functional recovery in AIS patients. Future studies should focus on validating these findings in controlled settings, further exploring biomarker responses, and elucidating the mechanisms underlying MSC-mediated recovery. Additionally, ongoing safety monitoring is crucial, especially to evaluate the incidence and causes of SAEs, ensuring the safe integration of UCMSC therapy into clinical practice.

## Supplementary Material

Supplementary tables.

## Figures and Tables

**Table 1:** Demographic Characteristics of Enrolled Participants

Demographic	Cohort 1 (n = 3)	Cohort 2 (n = 3)	Total (n = 6)
Age (years)			
Mean ± SD	66 ± 9.47	62 ± 11.68	64 ± 9.75
Range	(55.1 - 72.2)	(54.7 - 75.5)	(54.7 - 75.5)
Sex			
Famale (n, %)	2 (66.67%)	2 (66.67%)	4 (66.67%)
Body Weight (kg)			
Mean ± SD	58.1 ± 2.69	70.7 ± 7.85	64.4 ± 8.70
Range	(55.0 - 60.0)	(66.0 - 79.8)	(54.0 - 79.8)
Medical History (n, %)			
Hypertension	3 (100%)	3 (100%)	6 (100%)
Diabetic mellitus	2 (66.67%)	2 (66.67%)	4 (66.67%)
Dyslipidemia	3 (100%)	2 (66.67%)	5 (83.33%)
Atrial fibrillation	1 (33.33%)	1 (33.33%)	2 (33.33%)
Prior stroke	0 (0%)	1 (33.33%)	1 (16.67%)
NIHSS scores			
Median (IQR)	9 (1)	12 (12)	9.5 (3)
Range	(9-10)	(5-17)	(5-17)
mRS at 90 day			
Median (IQR)	4 (2)	1 (3)	3 (3)
Range	(2-4)	(1-4)	(1-4)

kg = kilograms; SD = standard deviation; NIHSS = National Institutes of Health Stroke Scale; IQR = interquartile range; mRS = modified Rankin Scale.

**Table 2:** Overall Summary of AEs and TEAEs

Characteristic, n (%)	Cohort 1 (n = 3)	Cohort 2 (n = 3)	Total (n = 6)
Patients with AEs	2	3	5
Patients with TEAEs	2	3	5
Patients with SAEs	2	2	4
TEAE Severity* (%)			
Moderate	33.33%	33.33%	33.33%
Severe	0	33.33%	16.67%
Life-threatening	33.33%	33.33%	33.33%
TEAE Relationship* (%)			
Unlikely	66.67%	0	33.33%
Unrelated	0	100%	50.00%

AE = adverse event; TEAE = treatment-emergent adverse event; SAE = serious adverse event. * Calculated by the most severe TEAE or the highest probability that occurred to a subject.

**Table 3:** Number of Patients with SAEs by SOC and PT

System Organ Class Preferred Term (n)	Cohort 1 (n = 3)	Cohort 2 (n = 3)	Total (n = 6)
Infections and infestations (n)	1	2	3
COVID-19	0	2	2
Empyema	1	0	1
Infection	1	0	1
Pneumonia	1	0	1
Septic shock	1	0	1
Nervous system disorder (n)	1	1	2
Seizure	1	0	1
Vocal cord paralysis	0	1	1
Respiratory, thoracic and mediastinal disorders (n)	1	1	2
Acute respiratory distress syndrome	1	0	1
Respiratory failure	0	1	1

SAE = serious adverse event; SOC = system organ class; PT = preferred term.
