# Supplementary material for: A Phase I, Open-Label, Dose-Escalation Study to Evaluate the Safety and Tolerability of Intravenous UMC119-06 in Patients with Acute Ischemic Stroke
Source: Int J Med Sci. 2026 Jan 1;23(2):576–84. doi: 10.7150/ijms.124255 (PMC12825123; doi:10.7150/ijms.124255)
Supplement: Supplementary file 1 — Supplementary tables. [file ijmsv23p0576s1.pdf]

Supplemental Appendix

Table S1. Inclusion and Exclusion Criteria

Inclusion Criteria	Exclusion Criteria
1. Patients of age between 20 through 80 years.	1. Patients with occurrence of a hemorrhagic transformation of ischemic stroke as evidenced by CT.
2. Patients had an onset of ischemic stroke within 48 to 168 hours before start of treatment.	2. Patients with a lacunar a lesion of ≤ 1.5 cm of longest diameter or a brainstem infarct on MRI.
3. Patients with ischemic stroke with speech deficit documented by NIHSS of 5 to 20, not change by ≥ 4 points from baseline.	3. Patients with reduced level of consciousness (score of 3 for item 1a of NIHSS)
4. Patients had ischemic stroke with large-artery atherosclerosis or cardioembolism.	4. Patients experienced seizures since the onset of ischemic stroke.
5. Patients confirmed of hemispheric cortical infarct with brain MRI including imaging demonstrating an acute lesion < 100 mL.	5. Patients with significant head trauma (GCS = 3~8) or prior stroke within previous 3 months (except TIA).
6. Patients' mRS of 0 or 1, reported by subject or family, prior to the onset of symptoms of the current stroke.	6. Patients had uncontrolled hyper-tension despite antihypertensive treatments.
7. Patients with body weight of 50 to 90 kgs.	7. Patients with blood glucose concentration < 50 mg/dL or > 400 mg/dL.
8. Patient or legal guardian is willing to provide written ICF to participate in the study, and had discussion with the investigator or designee.	8. Patients with uncorrected coagulopathy.
9. Women of child-bearing potential should have a negative urine pregnancy test prior to administration of IP.	9. Patients with history of any type of malignancy.
	10. Patients with major surgery within 30 days.
	11. Patients are pregnant (or plan to become pregnant within 3 months of IP) or lactating.
	12. Patients have significant illness as judged by PI.
	13. Patients have the following conditions in laboratory tests.
	14. Patients are known to be HIV infection.
	15. Patients not have CT or MRI test.
	16. Patients unable to return in follow-up visits for clinical evaluation, laboratory studies, or imaging evaluation.
	17. Patients participated in another clinical study of new investigational therapies.
	18. Patients have the following medical history.

Table S2. Summary of AEs and TEAEs (by Event)

Characteristic (n)	Cohort 1	Cohort 2	Total
**Number of AEs**	23	17	40
**Number of TEAEs**	18	17	35
**Number of SAEs**	6	4	10
**DLT (n)**			
**Yes**	0	0	0
**No**	18	17	35
**TEAE Relationship (n, %)**			
**Mild**	3 (16.67%)	2 (11.76%)	5 (14.29%)
**Moderate**	9 (50.00%)	11 (64.71%)	20 (57.14%)
**Severe**	2 (11.11%)	3 (17.65%)	5 (14.29%)
**Life-threatening**	4 (22.22%)	1 (5.88%)	5 (14.29%)
**TEAE Relationship (n, %)**			
**Unlikely**	13 (72.22%)	0	13 (37.14%)
**Unrelated (n, %)**	5 (27.78%)	17 (100%)	22 (62.86%)
**TEAE Action taken – IP (n, %)**			
**Negative**	1	1	2
**Not Applicable**	2	2	4
**TEAE Action taken – Other (n, %)**			
**None**	4 (22.22%)	2 (11.76%)	6 (17.14%)
**Non-study treatment given**	14 (77.78%)	15 (88.24%)	29 (82.86%)
**TEAE outcome (n, %)**			
**Resolved**	8 (44.44%)	12 (70.59%)	20 (57.14%)
**Ongoing**	10 (55.56%)	5 (29.14%)	15 (42.86%)

AE = adverse event; TEAE = treatment-emergent adverse event; SAE = serious adverse event; DLT = dose-limiting toxicity; IP = investigational product.

Table S3. Statistical Analysis of mRS Scores

	Visit	Screening	5	6	7	8	9	10	11
	**Day**	**-7 ~ -1**	**14**	**30**	**90**	**180**	**270**	**360**	**450**
	**Patient 1**	0	5	4	4	4	4	3	3
**Cohort 1**	**Patient 2**	4	4	4	Withdrawn				
	**Patient 5**	4	3	2	1	0	0	0	0
	**Patient 6**	4	3	2	1	0	1	1	0
**Cohort 2**	**Patient 7**	4	5	4	4	4	4	4	4
	**Patient 8**	5	4	3	1	1	1	1	1
**N**		6	6	6	5	5	5	5	5
**Mean**		3.5	4	3.2	2.2	1.8	2	1.8	1.6

Table S4. Statistical Analysis of BI Scores

Visit		Screening	5	6	7	8	9	10	11
Day		-7 ~ -1	14	30	90	180	270	360	450
Cohort 1	Patient 1	10	20	20	20	20	20	25	30
	Patient 2	0	20	20	0	Withdrawn			
	Patient 5	35	40	75	85	95	95	100	100
Cohort 2	Patient 6	35	60	75	85	95	85	85	90
	Patient 7	0	0	10	-	20	20	20	20
	Patient 8	0	35	50	85	95	95	95	95
N		6	6	6	5	5	5	5	5
Mean		13.33	29.16	41.67	55.0	65.0	63.0	65.0	67.0
Mean change from baseline		-	15.83	28.33	41.67	51.67	49.67	51.67	53.67
P value		-	0.063	0.031*	0.125	0.063	0.063	0.063	0.063

P-value was calculated using by Wilcoxon signed-rank test. *: statistical significance

Table S5. Statistical Analysis of NIHSS Scores

Visit	Day	Cohort 1			Cohort 2			Mean	Mean Change from Baseline	P value
		Patient 1	Patient 2	Patient 5	Patient 6	Patient 7	Patient 8			
**Screening**	**-7 ~ -1**	10	9	9	5	12	17	10.33	-	-
**1**	**0**	10	11	8	5	14	17	10.83	0.5	0.5
**2**	**1**	11	11	6	2	14	17	10.17	-0.2	0.75
**3**	**3**	9	11	5	2	14	15	9.33	-1	0.438
**4**	**7**	9	12	4	2	14	15	9.33	-1	0.563
**5**	**14**	9	11	3	2	14	12	8.5	-1.8	0.313
**6**	**30**	9	9	1	0	13	10	7.0	-3.3	0.188
**7**	**90**	9	17	0	0	-	5	6.2	-3.8	0.313
**8**	**180**	9	Withdrawn	0	0	9	3	4.2	-6.4	0.063
**9**	**270**	9		0	0	9	2	4.0	-6.6	0.063
**10**	**360**	9		0	0	9	2	4.0	-6.6	0.063
**11**	**450**	8		0	0	9	2	3.8	-6.8	0.063

P-value was calculated using by Wilcoxon signed-rank test.

Table S6. Statistical Analysis of MRI assessment

	Visit	Screening	6	7	11
	**Day**	**-7 ~ -1**	**30**	**90**	**450**
	**Patient 1**	68	11	0	0
**Cohort 1**	**Patient 2**	69	0	Withdrawn	
	**Patient 5**	0	0	0	0
	**Patient 6**	8	0	0	0
**Cohort 2**	**Patient 7**	14	0		
	**Patient 8**	55	0	0	0
**N**		6	6	4	4
**Mean**		35.67	1.83	0	0
**Mean change from baseline**		-	-33.84	-35.67	-35.67
**P value**		-	0.063	0.25	0.25

P-value was calculated using by Wilcoxon signed-rank test.
